# Impact of living and working in the heat on cognitive and psycho-physiological responses in outdoor fly-in fly-out tradesmen: a mining industry study

**DOI:** 10.3389/fphys.2023.1210692

**Published:** 2023-07-12

**Authors:** Sarah M. Taggart, Olivier Girard, Grant J. Landers, Ullrich K. H. Ecker, Karen E. Wallman

**Affiliations:** ^1^ School of Human Sciences, The University of Western Australia, Crawley, WA, Australia; ^2^ School of Psychological Science, The University of Western Australia, Crawley, WA, Australia

**Keywords:** heat stress, dehydration, mining industry, thermal strain, work

## Abstract

**Objective:** This study aimed to evaluate complex cognitive function, manual dexterity and psycho-physiological parameters in tradesmen working outdoors in the mining industry during summer and winter.

**Methods:** Twenty-six males working in a mining village in the north-west of Australia were assessed pre- and post-an 11-h shift at the start, middle, and end of a 14-day swing in summer (average daily temperature: 33.9°C, 38% RH; *n* = 12) and winter (24.3°C, 36% RH; *n* = 14).

**Results:** Working memory performance did not differ between seasons, over the swing or shift (*p* ≥ 0.053). Processing efficiency and manual dexterity performance did not differ between seasons (*p* ≥ 0.243), yet improved over the course of the swing (*p* ≤ 0.001) and shift (*p* ≤ 0.001). Core temperature, heart rate, thermal comfort, rating of perceived exertion and thermal sensation were not significantly different between seasons (*p* ≥ 0.076); however, average shift dehydration was greater in winter compared to summer (1.021 ± 0.005 vs. 1.018 ± 0.006; *p* = 0.014).

**Conclusion:** The ability to self-regulate the intensity of activity likely helped outdoor workers to thermoregulate effectively, minimising thermal strain during their swings and shifts, in turn explaining unaltered cognitive function and manual dexterity performance between seasons. Regardless of season, workers should receive education on dehydration and workplace risks to protect their health.

## Introduction

The expansion of the mining industry worldwide has led to a greater number of people working away from home as fly-in fly-out (FIFO) employees (Constable, 2022). In Australia, mining sites are typically located in remote areas, requiring workers to reside there for one to six weeks [known as a swing (Joyce et al., 2013);]. A challenge to working on mine sites often relates to the hot climate, which can elevate thermal strain. Sustained exposure to these hostile conditions while conducting physical labour can result in fatigue (Riethmeister et al., 2018), and a decline in physical work capacity (Foster et al., 2021) and cognitive function (Hancock and Vasmatzidis, 2003) over the course of a work shift.

Hot environmental working conditions can result in dehydration, with studies evaluating miners working in hot climates reporting urinary specific gravity (USG) values > 1.020, indicating that workers commenced work in a dehydrated state (Brake, 2001; Brake and Bates, 2003). Importantly, Brake et al. (1998) reported that dehydration of 1%–2% and 3%–4% of body-mass resulted in reductions in physical work rate ranging from 6%–7% and 22%–50% in moderate and hot environments, respectively. When dehydrated to 2% of body-mass, cognitive and motor performance decline rapidly at higher levels of hyperthermia (core temperature [T_c_] = 39.5°C), compared to moderate levels (T_c_ = 38.5°C) (Piil et al., 2017).

Cognitive impairment can increase the risk of workplace injuries (Gaoua et al., 2011), thus alterations in complex cognitive performance due to heat exposure is an important consideration for outdoor workers. Gaoua et al. (2011) evaluated cognitive performance, following 60-min of walk/rest in hot (50°C, 50% relative humidity [RH]) and cool conditions (20°C, 40% RH). Mean T_c_, in the heat increased to hyperthermic levels (38.6°C vs. 37.1°C in cool conditions), with this resulting in impairment in complex task performance.

Many tradesmen, including those in the mining industry, need to possess fine motor skills and coordination to ensure optimal performance (Agha et al., 2015; Valenza et al., 2020). Valenza et al. (2020) assessed simple and complex manual dexterity performance in seven males prior to and after 60 min of exposure to either hot (37°C, 40% RH), neutral (21°C, 40% RH) or cold (7°C, 40% RH) environments, whilst participants were mentally fatigued (induced by a 35-min cognitive battery test) or not (no cognitive testing). When considering temperature alone, complex task performance deteriorated by 16% in heat, compared to neutral conditions, with larger impairment occurring (36%) when participants also experienced mental fatigue in the heat. Additionally, complex task performance was impaired when participants experienced mental fatigue in neutral conditions (decline of 36%). The impact of heat and mental fatigue on dexterity skills is important to consider/monitor so to avoid workplace accidents.

Exposure to hot-humid conditions for consecutive days can lead to physiological adaptations (known as “heat acclimatisation”), which can result in increased tolerance to high ambient temperatures (Periard et al., 2021). These adaptations include increased sweat-rate, lower resting and exercise T_c_ and heart rate (HR), lower sweating thresholds, greater skin and muscle blood flow and a greater fluid electrolyte balance (Sawka et al., 2011). With many mining workers spending between 8-21 consecutive days on site working under heat stress, it is possible that acclimatisation can occur. Whether acclimatisation has occurred over a swing is difficult to determine as previous mining studies have focused on assessing psycho-physiological and/or cognitive variables over the course of a shift at only one time point throughout the swing (Peiffer and Abbiss, 2013; Polkinghorne et al., 2013; Riethmeister et al., 2019). To date, no studies have quantified the effect of prolonged heat exposure on complex cognitive function, manual dexterity, fatigue, and physiological parameters on multiple days over a swing.

This study aimed to investigate the effects of season (summer vs. winter) on cognitive and psycho-physiological responses, over the course of three 11-h shifts assessed at the start, middle and end of a 14-day swing in tradesmen working outdoors in a mine site village. We hypothesised that over the course of a swing and shift i) fatigue and dehydration would worsen; ii) cognitive function and manual dexterity performance would deteriorate as workers became more fatigued; iii) thermal strain would increase during shifts yet decrease over the swing due to acclimatisation (in summer only); iv) and that all of the above changes would be accentuated in summer than winter.

## Methods

### Participants

A cohort of 26 males [summer: *n* = 12 (grounds = 3, electricians = 2, plumbers = 2, carpenters = 2, refrigeration technicians = 3); winter: *n* = 14 (grounds = 4, electricians = 2, plumbers = 2, carpenters = 3, refrigeration technicians = 3)] volunteered for this study. All participants were tradesmen who could work at a self-paced rate. Characteristics between seasons did not differ (summer: age = 46 ± 14 y, BMI = 29.1 ± 5.7 kg/m^2^, length of employment = 2.1 ± 1.9 y; winter: age = 44 ± 12 y, BMI = 31.2 ± 4.1 kg/m^2^, length of employment = 1.6 ± 1.7 y). Participants were FIFO employees who were regularly flown into a mine site village in the north-west of Australia to work for 14 days continuously (swing) on site and then flown home (all residing in a 2 h radius from Perth region) for a 7-day break. All participants were informed about the requirements of the study before providing informed consent. Ethics approval was granted by the Human Research Ethics Committee of the University of Western Australia (RA/4/20/6536).

### Study design

Workers were assessed in respect to cognitive function, manual dexterity performance, and psycho-physiological variables over the course of a shift in hot (March in summer; average ambient temperature: ∼33.9°C [range: 21.4°C–43°C]) and temperate (July in winter; average temperature: ∼24.3°C [range: 6.8°C–32.7°C]) environments. Three of the participants were tested during both summer and winter months. Participants underwent a familiarisation session on day 1 of their swing and were then tested at the start (days 2/3), middle (days 7/8) and end (days 12/13) of their 14-day swing. During their 11-h shift, participants were tested at the start (6–7 a.m.) and end (5–6 p.m.) of the day. Participants wore steel cap boots, yellow-high visibility long sleeve shirt, navy trousers and a hat for each session. A food and fluid consumption diary was completed during the 11-h shift.

### Familiarisation session

Demographic and anthropometric information was recorded by participants. Participants were introduced to all the physiological equipment and perceptual scales. They also performed the manual dexterity and cognitive tasks (counting span task) five times each in order to reduce any potential learning effect (Saldaris et al., 2019).

### Protocol

Upon arrival to work, participants were fitted with HR monitors and accelerometers. They provided a urine sample 30-min prior to the start (and end) of their shift to assess for USG. Afterwards, they attended a ∼25 min pre-work meeting where they were assigned daily tasks. At the meeting conclusion, participants completed baseline testing (see “testing during work-shift”), which was conducted outdoors in a seated position. The baseline test battery was replicated at the end-of-shift. Additionally, T_c_, HR, thermal sensation, thermal comfort and rating of perceived exertion (RPE) were measured at midday. Only peak values for each of these variables are presented, with the exception of T_c_ which also has baseline values presented. Throughout the shift, participants conducted a broad range of tasks (i.e., digging, carrying loads, driving, gardening, working with tools, etc.) predominantly outdoors (∼80%).

### Testing during work-shift

Participants rotated through various testing stations in order to assess: (1) manual dexterity performance and cognitive function, (2) fatigue and depression, anxiety and stress and (3) HR, T_c_ and perceptual measures of thermal sensation, thermal comfort and RPE. The Depression, Anxiety and Stress Scale (DASS) was administered only pre-shift. Tests were performed in the same order for a given participant in all their testing sessions. Measures of environmental conditions [wet bulb, ambient temperature, globe temperature, wet bulb globe temperature (WBGT) and RH] were monitored hourly via the QuesTEMP 32 (TSI Incorporated, United States; accuracy±0.5°C), while wind speed was also measured at similar intervals via a digital anemometer (Model:AM-4203HA, Lutron Electronic Enterprise Co., LTD., Taiwan; accuracy 0.1± km/h). Environmental values were averaged across the course of the shift for analysis.

### Physiological responses

Core temperature was recorded via an ingestible radio-telemetric thermistor using a CorTemp Data Recorder 262 K device (CorTemp HQ Inc., Palmetto, United States; accuracy±0.1°C). Heart rate was measured continuously via a chest based polar monitor throughout the work-shift (Polar H7, Finland). Participants wore accelerometers (Actigraph GT3X, Pensacola, United States) on their hip. Data recorded continuously (epoch 30 Hz) during the shift and was downloaded using ActiLife (Actilife, version 6.13.4, Pensacola, United States). A hand-held refractometer (ATAGO Model URC-N_E_, Japan) measured USG. Values for USG were defined as “*well hydrated*” <1.010, “*minimal dehydration*” 1.010–1.020, “*significant dehydration*” 1.021–1.030 and “*serious dehydrated*” >1.030 (Casa et al., 2000).

### Perceptual responses

Thermal sensation (0 [very cold] to 20 [very hot]) and thermal comfort (0 [very comfortable] to 20 [very uncomfortable]) were recorded using visual analogic scales ranging from green to red and white to black, respectively, (Gaoua et al., 2012). Higher thermal sensation and thermal comfort scores represented feeling hotter and less comfortable, respectively. Ratings of perceived exertion (RPE; 6 [no exertion at all] to 20 [maximal exertion]) were measured using the Borg scale (Borg, 1982).

### Fatigue and mental health

The Multidimensional Fatigue Scale has been previously validated in army recruits and junior doctors and demonstrated good internal consistency (Cronbach alpha = 0.84; Smets et al., 1995). This scale assesses physical, mental and general fatigue, reduced motivation and reduced activity. Briefly, it is scored on a 5-point scale (1 [yes, this is true] to 5 [no, this is not true]), with higher scores representing greater levels of fatigue/reduced motivation or activity, with each subsection made up of 4 questions. The Depression, Anxiety and Stress Scale is a self-report scale that measures negative emotional states and is assessed using a 4-point scale (0 [never] to 3 [always]), with each subsection made up of 7 questions. The short form version, which has demonstrated good construct validity and reliability in a non-clinical sample (Cronbach alpha = 0.82–0.93; Henry and Crawford, 2005), has previously been used in the Australian FIFO industry (Velander et al., 2010; Barclay et al., 2013).

### Manual dexterity and cognitive function

The Purdue pegboard task (Model 32020, J.A Preston Corporation, New York) was used to assess manual dexterity performance (i.e., concentration, fine motor skills and hand-eye coordination) (Palejwala et al., 2019). Working memory capacity was assessed using a modified version of the counting span task (Inquisit Lab 6, Millisecond Software, Seattle, United States) that requires ∼5 min to complete (Conway et al., 2005). This task requires participants to count the green dots on a sequence of cards containing yellow and green dots and then recall the counts in order, with set size ranging from 2 to 7. Counting latency, first recall latency, subsequent recall latency, recall latency, number of cards counted correctly, number of counts recalled correctly and counting span were recorded (Taggart et al., 2023). Individual counting and recall latency times (ms) were aggregated across all trials with the same number of target dots, or within the relevant serial position, respectively. Different number sequences were randomly assigned throughout the testing sessions.

### Statistical analysis

Data are expressed as mean ± standard deviation. Statistical analysis was conducted using R studio 1.4.1717. Mean values across a day were calculated for environmental conditions. Linear mixed model analysis was used to compare peak shift values (the highest value of the three time points) for T_c_ (and baseline values), HR, thermal sensation, thermal comfort and RPE, with season and swing included as fixed effects and participant as a random effect. An analysis was conducted for mean HR, however the results were similar to peak HR, these results were included as a supplementary data file. For cognitive function, manual dexterity performance, USG and fatigue, pre- and post-shift values were compared using a linear mixed model analysis with season, swing and shift included as fixed effects (and target dots counted for counting latency) and participant as a random effect. Where appropriate, post hoc comparisons using *Tukey LSD* were conducted. Statistical significance was accepted at *p* < 0.05. Cohen’s *d* effect sizes with ± 95% confidence intervals were calculated for primary variables within swing only (e.g., start of swing vs. middle of swing, etc.), with only moderate (*0.50–0.79*) to large (*>0.80*) effect sizes reported.

## Results

### Environmental conditions

Mean ambient temperature (33.9 ± 4.2 vs. 24.3°C ± 4.8°C; *p* < 0001), globe temperature (42.5 ± 7.4 vs. 31.5°C ± 8.4°C; *p* < 0.001) and WBGT (29.7 ± 2.8 vs. 20.3°C ± 4.2°C; *p* < 0.001) were significantly higher in summer (*n* = 18) compared to winter (*n* = 14). However, RH (38% ± 18% vs. 36% ± 20%; *p* = 0.450) and wind speed (9.3 ± 7.7 vs. 7.3 ± 5.3 km/h; *p* = 0.28) did not differ. There was no main effect of swing (*p* > 0.07) on any environmental parameter. Maximum ambient temperatures in summer and winter were 37.9°C ± 2.2°C and 28.8°C ± 2.3°C, respectively.

### Processing efficiency

#### Counting latency

There were significant main effects of swing, shift and number of target dots (*p* < 0.001) for counting latency. These were supported by significant interaction effects of season by shift (*p* = 0.003), swing by shift (*p* = 0.010), swing by number of target dots (*p* < 0.001) and shift by number of target dots (*p* = 0.004). Counting latency was significantly greater pre- compared to post-shift, but mainly in summer, mainly at the beginning (and middle) of a swing compared to the end, and mainly when counting more than seven dots (*p* ≤ 0.004). Counting latency was significantly longer at the beginning of the swing compared to the middle and end, but mainly when counting five or more dots (*p* ≤ 0.022). Counting latency tended to be longer at the start of a swing compared to the end (*d = 0.56* [-0.03, 1.07]).

#### Counting correct responses

There were no significant main or interaction effects for the number of counting correct responses (*p* ≥ 0.096). Mean correct responses were 42 ± 11 and 48 ± 8 in summer and winter, respectively.

### Working memory capacity

#### Recall latency

**FIGURE 1:**
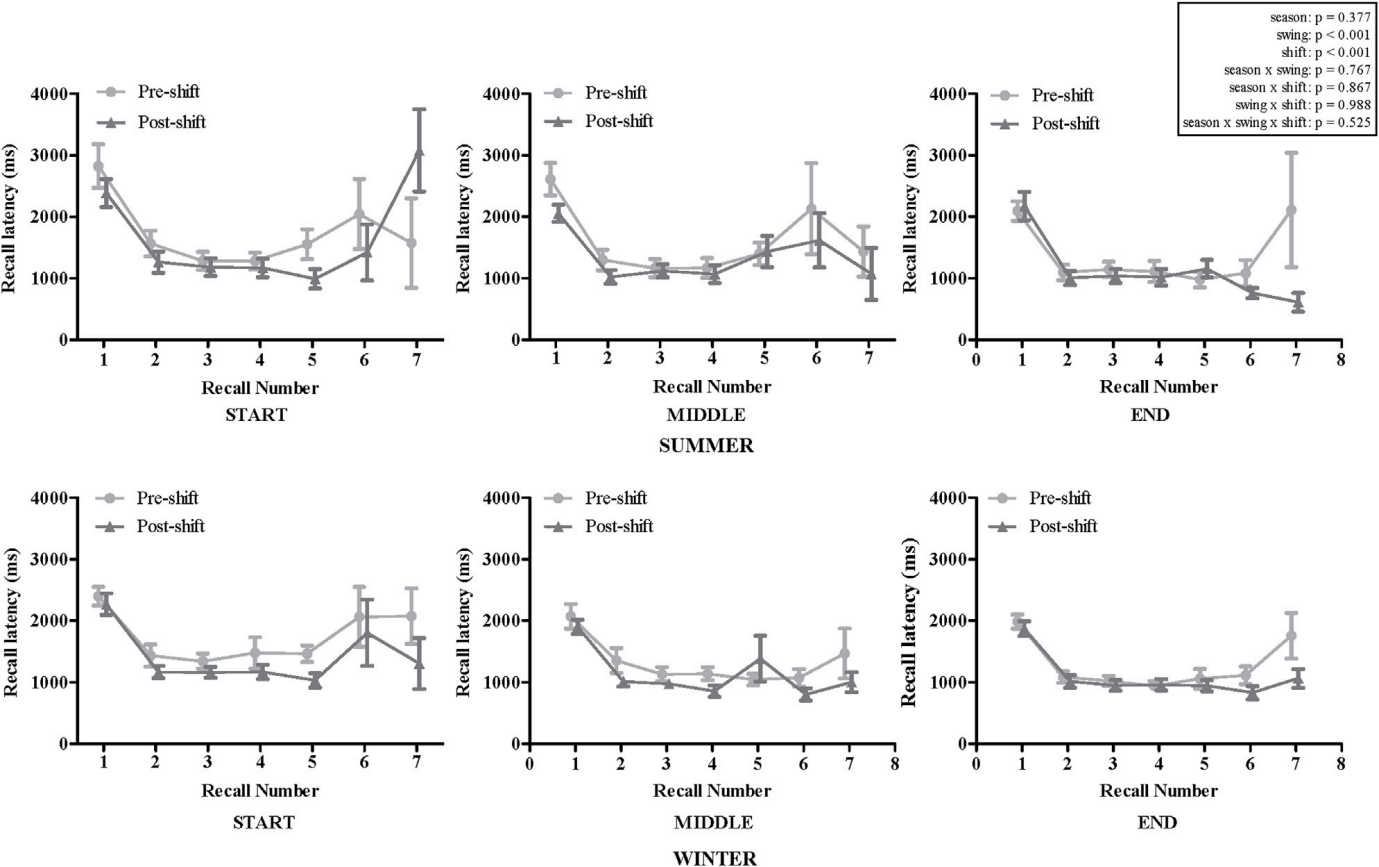
Mean recall latency (±SEM) pre- and post-shift at the start, middle and end of a swing in summer (*n* = 12) and winter (start and end of swing *n* = 14, middle of swing *n* = 12).

No significant interaction effects were observed for any variables or main effect of season. However, significant main effects occurred for swing and shift (both *p* < 0.001; Figure 1). Recall latencies were shorter in the middle (−13.2%; *p* = 0.017) and end (−21.2%; *p* < 0.001) compared to the start of the swing. Recall latencies were faster post-shift (−16.8%) compared to pre-shift. First response latency was significantly greater than subsequent response latency, regardless of season, swing or shift (*p* < 0.001). Mean first response latency was 2222 ± 744 ms compared to subsequent response latency of 1,269 ± 1,022 ms.

#### Recall correct responses

There was no main effect of season, swing or shift for recall correct responses (*p* ≥ 0.334), nor were there significant interactions (*p* ≥ 0.257). Mean correct recall responses in summer were 38 ± 11 compared to 42 ± 11 in winter.

#### Counting span

There were no main effects of season, swing or shift (*p* ≥ 0.179) nor were there significant interaction effects for counting span score between season, swing and/or shift (*p* ≥ 0.375). Mean counting span scores in summer were 5.22 ± 1.21 vs. 5.66 ± 1.27 in winter.

### Manual dexterity

There was no main effect of season for manual dexterity of the dominant hand (*p* = 0.243), however swing showed a significant main effect (*p* < 0.001). Compared to the start, manual dexterity significantly improved in the middle (*d = -0.50* [-1.02, 0.10]; *p* = 0.029) and at the end (*d = -0.50* [-1.01, 0.09]; *p* < 0.001) of the swing. There was a significant main effect of shift (*p* < 0.001; Table 1). There were no significant interactions between season, swing and/or shift (*p* > 0.05).

**TABLE 1:** Manual dexterity performance pre- and post-shift at the start, middle (winter: *n* = 12) and end of a swing.

	Dominant hand*^#^		Non-dominant hand*^#^	
	Pre-shift	Post-shift	Pre-shift	Post-shift
**Summer (*n* = 12)**				
Start of swing	15 ± 2	16 ± 2	14 ± 3	15 ± 3
Middle of swing	16 ± 2	16 ± 2	15 ± 3	15 ± 3
End of swing	16 ± 3	17 ± 2	15 ± 3	15 ± 2
**Winter (*n* = 14)**				
Start of swing	15 ± 2	16 ± 2	15 ± 2	16 ± 2
Middle of swing	16 ± 2	17 ± 2	16 ± 2	17 ± 2
End of swing	17 ± 2	18 ± 2	15 ± 2	16 ± 2

*, denotes a significant main effect for shift (*p* < 0.05).

#, denotes a significant main effect for swing (*p*< 0.05).

For the non-dominant hand, there were significant main effects of swing and shift (*p* < 0.001). Performance over the course of the swing improved, with scores in the middle (*d = -0.50* [-1.02, 0.10]; *p* = 0.010) and end (*d = -0.50* [-1.01, 0.09]; *p* < 0.001) of a swing significantly greater than scores at the start. For shift, performance post-was significantly better than pre-shift (*p* = 0.001: Table 1). There were no significant interactions between season, swing and/or shift (*p* > 0.05).

### Fatigue and mental health

There were no significant main effects of season, swing or shift for physical, mental or general fatigue, or activity (*p* > 0.05; Table 2). For motivation, there was a significant main effect of shift, where participants reported lower motivation (*p* = 0.032) post-compared to pre-shift. There was a significant interaction effect for physical fatigue between season and shift (*p* = 0.035).

**TABLE 2:** Mean fatigue scores pre- and post-shift at the start, middle (winter: *n* = 12) and end of a swing.

	General fatigue		Physical fatigue		Mental fatigue		Reduced motivation		Reduced activity	
	Pre-shift	Post-shift	Pre-shift	Post-shift	Pre-shift	Post-shift	Pre-shift	Post-shift*	Pre-shift	Post-shift
**Summer (*n* = 12)**										
Start of swing	8 ± 3	8 ± 3	8 ± 3	6 ± 3	8 ± 2	7 ± 3	6 ± 2	7 ± 2	8 ± 2	7 ± 3
Middle of swing	9 ± 4	8 ± 3	8 ± 4	6 ± 2	7 ± 3	7 ± 2	7 ± 3	7 ± 3	7 ± 3	8 ± 2
End of swing	8 ± 3	8 ± 3	7 ± 2	7 ± 3	7 ± 3	7 ± 3	6 ± 2	7 ± 3	7 ± 3	7 ± 3
**Winter (*n* = 14)**										
Start of swing	9 ± 4	9 ± 3	8 ± 4	9 ± 2	8 ± 3	8 ± 3	8 ± 3	8 ± 2	8 ± 4	9 ± 3
Middle of swing	9 ± 3	10 ± 3	9 ± 3	9 ± 3	9 ± 3	10 ± 3	8 ± 3	9 ± 3	9 ± 3	9 ± 3
End of swing	9 ± 4	9 ± 3	8 ± 3	8 ± 3	8 ± 3	8 ± 3	8 ± 3	8 ± 3	8 ± 3	9 ± 3

*, denotes a significant main effect for shift (*p* = 0.032).

Depression and anxiety did not show any significant main effects of season or swing, nor was there a significant interaction effect between season and swing for anxiety, stress or depression sub-scores. However, there was a significant main effect for stress for swing, whereby stress was greater at the start compared to the middle of the swing (*p* = 0.025). Anxiety (4.2 ± 5.2), depression (4.5 ± 5.5) and stress scores (7.1 ± 6.3) were categorised as “normal”.

### Physiological responses

#### Core temperature

**FIGURE 2:**
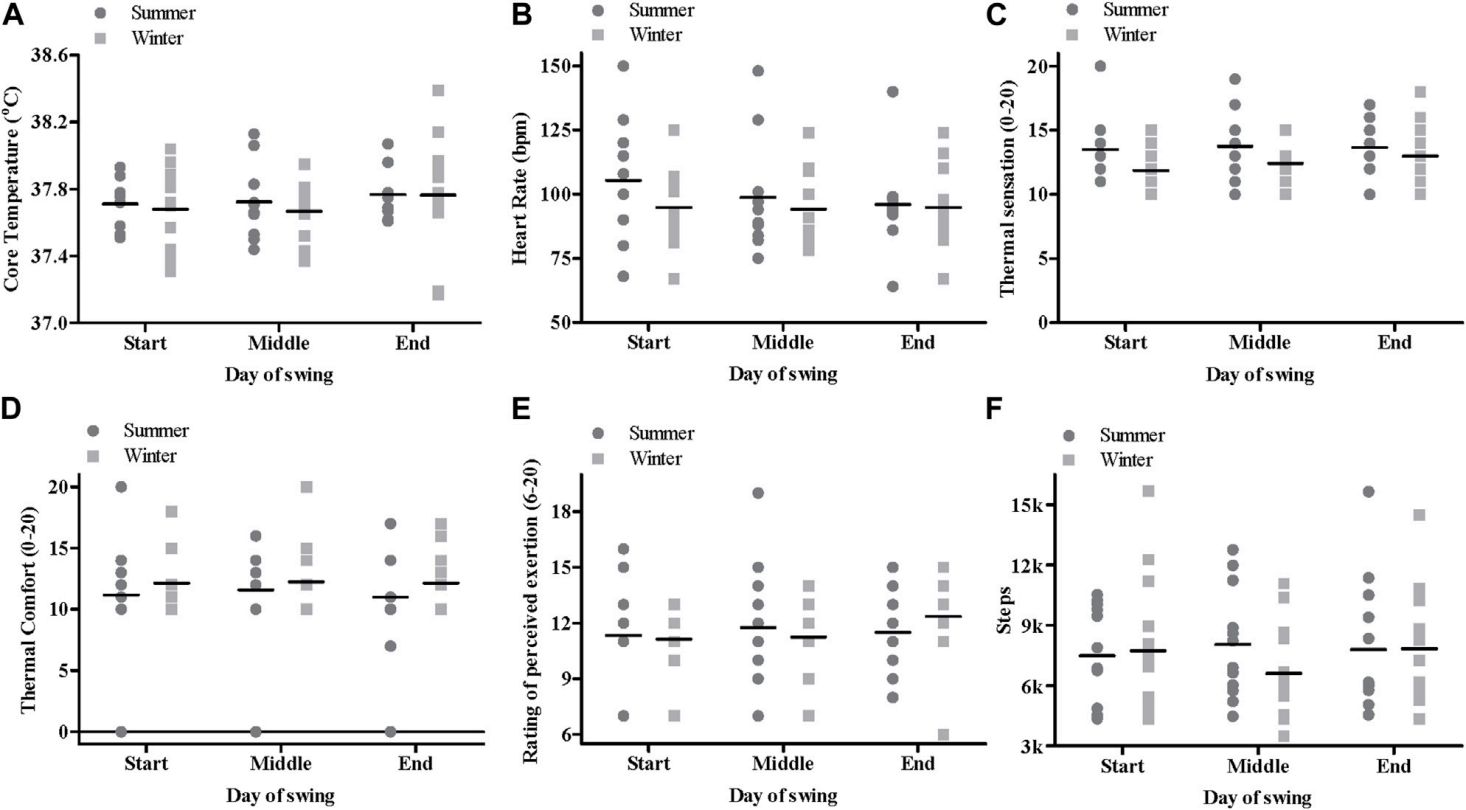
Mean and individual data for peak core temperature **(A)**, peak heart rate **(B)**, highest thermal sensation **(C)**, highest thermal comfort **(D)**, highest rating of perceived exertion values **(E)** and steps **(F)** in summer and winter at the start, middle and end of a swing.

Peak T_c_ occurring during an 11-h shift did not differ significantly between seasons (*p* = 0.768) or throughout the swing (*p* = 0.339) (Figure 2A). There was no interaction effect between season and swing (*p* = 0.904). Baseline T_c_ did not differ between seasons (*p* = 0.689) or throughout the swing (*p* = 0.608), with average baseline T_c_ in summer 37.26°C ± 0.28°C and winter 37.23°C ± 0.24°C. From pre-to post-shift T_c_ increased by 0.29°C and 0.26°C in summer and winter, respectively.

#### Heart rate

There was no significant difference between summer and winter (*p* = 0.351), nor was there a main effect for swing (*p* = 0.320; Figure 2B). No significant interaction effect between season and swing were observed for HR (*p* = 0.318).

#### Hydration

There was a significant main effect of season (*p* = 0.014), where USG in winter (1.021 ± 0.005) was significantly higher than summer (1.018 ± 0.006), irrespective of swing or shift (Table 3). There was no significant main effect of swing (*p* = 0.597) or shift (*p* = 0.602), nor was there any significant interaction effects. (*p* > 0.05). Total fluid consumption between summer (3.85 ± 1.50 L/day) and winter (3.45 ± 1.27 L/day) and throughout the swing did not differ (*p* = 0.305–0.379).

**TABLE 3:** Number of participants in each USG classification in summer (*n* = 12) and winter (start and end of swing *n* = 14, middle of swing *n* = 12) pre- and post-shift.

	< 1.010 “*well hydrated*”		1.010–1.020 “*minimal dehydration*”		1.021–1.030 “*significant dehydration*”		> 1.030 “*serious dehydration*”	
	Pre-shift	Post-shift	Pre-shift	Post-shift	Pre-shift	Post-shift	Pre-shift	Post-shift
**Summer^**								
Start of swing	1	2	8	3	3	7		
Middle of swing		2	7	2	5	8		
End of swing		1	9	6	3	5		
**Winter**								
Start of swing		2	5	3	8	9	1	
Middle of swing			8	5	4	7		
End of swing		1	8	5	5	8	1	

^, denotes a significant main effect for season (*p* < 0.05).

#### Activity

There was no significant main effect of season (*p* = 0.888) or swing (*p* = 0.919) for steps counted. There was no significant season by swing interaction (*p* = 0.609). Mean steps count in winter was 7429 ± 2890 and in summer was 7774 ± 2821.

### Perceptual responses

There was no season by swing interaction (*p* ≥ 0.383) nor any main effects of season (*p* ≥ 0.076) or swing (*p* ≥ 0.352) for thermal sensation, thermal comfort and RPE (Figure 2C–E).

## Discussion

### Cognitive function

Working memory and processing efficiency were not different between seasons, contradicting our hypothesis that cognitive function would decline in summer compared to winter due to exacerbated thermal strain, fatigue and dehydration. These observations are supported by Girard et al. (2021) who assessed cognitive function and T_c_ responses over a shift in oil and gas workers in summer (41°C) and winter (17°C) in the Middle-East. These researchers reported no difference between seasons for recognition memory, working memory and executive function tasks. However, all testing by Girard et al. (2021) was conducted indoors in a quiet room, which may have contributed to greater focus and attention on the task. Conversely; Saini et al. (2017) observed that when soldiers were tested in a hot desert (42°C) compared to temperate (27°C) conditions, performance on cognitive tasks requiring attention, concentration, delayed and immediate recall, visual retention, and recognition were impaired. However, these authors did not assess T_c_ or perceptual variables, making it difficult to determine the exact reasons for deteriorated cognitive function with heat exposure. Lack of change in cognitive function between seasons in the current study may be due to the ability of workers to self-pace their work. Self-pacing (behavioural thermoregulation) by workers may also explain the absence of a clinically significant elevation in T_c_ (>38.5°C (Taylor et al., 2016)) throughout the shift. While there was no difference in the total number of steps recorded between seasons, it is possible that workers adjusted their pace to accommodate the tasks and/or took more frequent rest breaks. This adaptive behaviour may have facilitated effective thermoregulation, as evidenced by comparable T_c_ and HR values observed in both seasons. Another explanation may relate to dehydration levels not being ≥2% loss of body-mass during the summer months, with losses of this magnitude found to impair working memory performance (Gopinathan et al., 1988). However, this is speculative as only changes in USG were assessed and not body-mass.

Over the course of a swing, both recall and counting latency significantly improved, regardless of season. This result was unexpected and did not support our hypothesis that cognitive function would deteriorate throughout a swing. This result may be related to the sudden change in lifestyle/schedule when workers returned to site for work. Many workers spend their week off between swings resting, hence the return to work which involves adapting to an early-wake up sleep routine and the general workplace environment at the start of a swing may have negatively affected initial processing efficiency. Asare et al. (2022) conducted a survey of 216 Australian FIFO workers and reported that total sleep time on site was significantly less compared to when at home. If sleep time or attention increased over the course of the swing then this may be a reason for improved speed in respect to recall and counting latency. Another possible explanation for improvement in latency could be attributed to a practice/learning effect (Tao et al., 2019), as participants had to execute the task on nine occasions. However, we were careful to include five practice trials during the familiarisation process in an attempt to address this issue.

Although correct responses or counting span scores did not change over the shift, recall and counting latency improved. This may be partially explained by the body’s circadian rhythm, as the ability to perceive stimuli, as well as the response speed to a task, is faster in the afternoon compared to morning (Wright Jr et al., 2002). Another explanation could relate to the early morning start, where workers may have felt tired and less alert from just waking up (Valdez, 2019).

### Fatigue

Perceived fatigue did not differ between seasons, throughout the swing or over the course of a shift. This contradicted our hypothesis that fatigue would be greater at the end of a swing and shift. This finding challenges previous research investigating subjective fatigue in FIFO workers (Muller et al., 2008; Riethmeister et al., 2018). Muller et al. (2008) assessed occupational fatigue and vigilance at a remote mine site in Australia where ambient temperatures were hot all year around (values not reported). Worker duties ranged from routine maintenance tasks to control of data management screens. Workers reported significantly greater fatigue from day-shift 8 onwards, and at the end of night-shifts 1-3 compared to start of night shifts (Muller et al., 2008). Inconsistencies in findings between the current study and Muller et al. (2008) are hard to determine due to the lack of measurement of physiological values, as well as the absence of detail around work-rate. Further, Bazazan et al. (2019) assessed perceived fatigue in 287 male shift workers in the petrochemical industry in Iran during April [∼32°C] and September [∼41°C (Weather Spark, 2023);]. General fatigue was found to be perceived as high, with moderate elevations in physical and mental fatigue levels. Discrepant findings between Bazazan et al. (2019) and the current study may relate to methodological differences such as: i) differing working rosters and times of shifts between studies, ii) difference in ambient temperatures, iii) and/or dehydration experienced by participants [not reported by Bazazan et al. (2019)]. These factors could have, together or alone, influenced thermal strain, and hence fatigue, experienced by workers. Another explanation for the lack of change in perceived fatigue scores between seasons may be due to workers being able to self-pace their work intensity in response to perceptual cues related to sensations of fatigue. Further research should investigate the relationship between heat exposure on FIFO workers’ fatigue, sleep, and cognitive function.

### Hydration

Contrary to our hypothesis, dehydration levels were significantly higher in winter (USG = 1.021), classifying workers are “*significantly dehydrated*” compared to summer (USG = 1.018) where workers were classified as *“minimally dehydrated”* (Casa et al., 2000). These results may be due to the lack of stimulus to consume fluids throughout and after a shift in winter (i.e., lower thermal sensation scores in winter) and/or the possible reduced emphasis and education to drink in cooler conditions (Leiper, 2013). However, as we did not record fluid consumption pre- or post-shift we are unable to confirm this. Another explanation could relate to workers in winter potentially taking less/shorter unscheduled rest breaks during or after the completion of a task due to lower perceptions of thermal stress. In order to reduce dehydration and thermal strain, workers should be educated on the importance of appropriate hydration throughout their work shifts.

### Limitations

While no significant differences in participant demographic and anthropometric characteristics were observed between seasons, recruiting different workers for each season may have influenced the results. Factors such as individual heat and fatigue tolerance as well as cognitive task performance could vary among individuals, potentially impacting the outcomes. Additionally, a limited number of workers were available for recruitment due to the in-field nature of this study, hence the study may be underpowered due to limited sample availability. The fact that productivity, total body movement (particularly upper limb movement) and rest breaks were not assessed to determine the use of pacing strategies is also a limitation. Lastly, recording the amount of fluid (and alcohol) consumed pre-, during and post-shift may have helped further explain the difference in seasonal hydration status of workers and the influence this has on cognitive function. Future research should aim to assess this in mining workers so to better understand the impact of hydration strategies.

## Conclusion

This field study is the first to assess a wide range of parameters over three 11-h shifts across a 14-day swing in FIFO workers in Australia during summer and winter. Heat exposure during the summer months did not impair complex cognitive function, manual dexterity performance or accentuate fatigue throughout a swing for workers who were able to self-pace in their work duties. These results suggest that behavioural thermoregulation may play an important role in regulating physiological and cognitive responses when working in hot ambient conditions. Future studies should assess workers that do not have the ability to modify their work pace in order to determine whether heat stress deteriorates their productivity and cognitive function.

## Data Availability

The raw data supporting the conclusion of this article will be made available by the authors, without undue reservation.

## Abbreviations

T_c_, Core temperature; FIFO, Fly-in fly-out; HR, Heart rate; RPE, Rating of perceived exertion; RH, Relative humidity; USG, Urinary specific gravity; WBGT, Wet bulb globe temperature.
