# Supplementary material for: Impact of living and working in the heat on cognitive and psycho-physiological responses in outdoor fly-in fly-out tradesmen: a mining industry study
Source: Front Physiol. 2023 Jul 12;14:1210692. doi: 10.3389/fphys.2023.1210692 (PMC10368878; doi:10.3389/fphys.2023.1210692)
Supplement: Supplementary file 1 [file Table1.docx]

|  | **Start of swing** | **Middle of swing** | **End of swing** |
| --- | --- | --- | --- |
| **Summer** | 89±14 | 86±14 | 83±15 |
| **Winter** | 81±9 | 81±10 | 79±8 |

**Table S1.** Mean heart rate (bpm) in summer and winter over the course of an 11-h shift on each day of the swing.
